# Supplementary material for: The confidence-noise confidence-boost (CNCB) model of confidence rating data
Source: PLoS Comput Biol. 2025 Apr 21;21(4):e1012451. doi: 10.1371/journal.pcbi.1012451 (PMC12043244; doi:10.1371/journal.pcbi.1012451)
Supplement: S1 File — (PDF) [file pcbi.1012451.s001.pdf]

S1 File

The Confidence-Noise Confidence-Boost (CNCB) model of confidence rating data

Pascal Mamassian and Vincent de Gardelle

In this Supporting Information, we take three examples of confidence rating studies from the literature and apply the CNCB model described in the main text.

A. Bang et al. (2019; Experiment 2, Task 1)

The first example is a study by Bang et al. [1]. In one of the experiments (Experiment 2, Task 1), the authors studied how well participants performed over the course of a block of trials both in their perceptual decisions and their confidence judgments. For each trial, participants (N=159) were first asked to make a coarse orientation discrimination of a noisy Gabor patch (“left” or “right” tilt) and then a confidence rating on a 4-point scale. The original analysis was performed for each of the 97 trials of a block, but following our recommendations to infer reliably the confidence boost parameters, here we have grouped the trials in bins of 10 trials. Participants get better in the perceptual task (a form of perceptual learning, **Figure 1A**), but, interestingly, they simultaneously get worse in evaluating their own performance as shown by the decreasing CNCB efficiency (**Figure 1C**). This result correlates very well with the original finding that used the M-ratio as a measure of confidence efficiency (**Figure 1D**).

The CNCB framework allows us to go one step further and to estimate confidence noise and confidence boost in this experiment. The CNCB model fits the data well for the blocks of trials. For instance, for the group of trials in the middle of the block (trials 38-47), the analysis of the Deviance showed that the model fit was not statistically different from the best possible fit (chi2(11) = 17.6, p = 0.09; **Figure 1B**). Looking at confidence noise, we find that it decreases with trial number within a block (**Figure 1E**), thus explaining the decrease in confidence efficiency. Interestingly, there also seems to be an increase of confidence boost, especially at the beginning of the experiment (**Figure 1F**). This suggest that over the blocks, participants actually did learn to use some information about the stimulus above and beyond what they used to reach their perceptual decision. This increase in confidence boost should have improved their confidence efficiency, but it seems that the increase in confidence noise was even larger, so overall confidence efficiency decreased. Of course, the experiment from Bang et al. [1] was not designed to be analysed in terms of confidence noise and confidence boost, so any re-interpretation of their data is purely speculative.

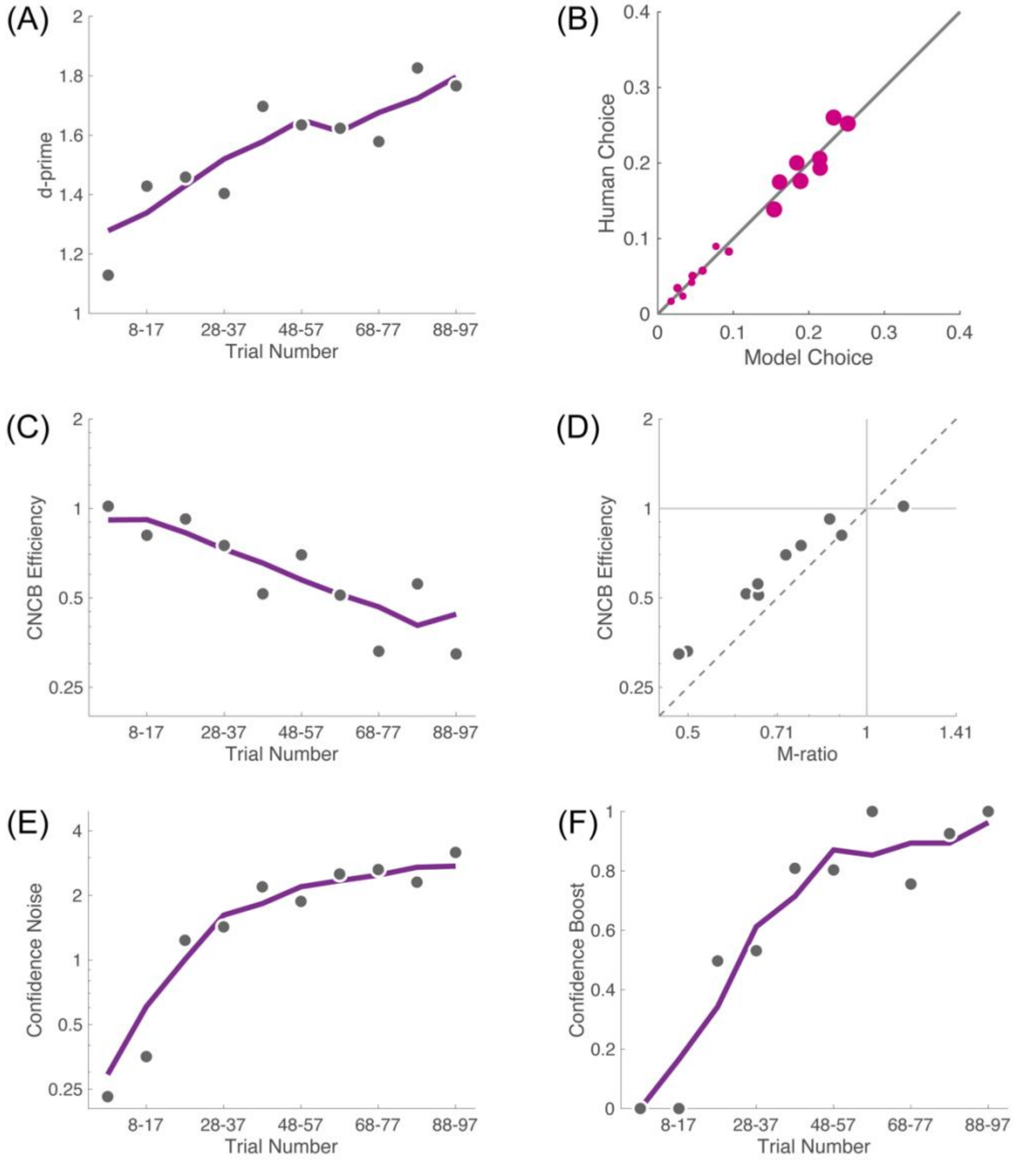

Figure 1. CNCB analysis of Bang et al. [1], Experiment 2, Task 1 (coarse discrimination). **(A)** Perceptual sensitivity (d') is increasing over the block of trials. Each dot corresponds to the sensitivity for a group of 10 trials (except the first bin that contains only 7 trials) computed by pooling all observers. The solid line is the running average over 3 consecutive groups of trials. **(B)** Goodness of fit of the CNCB model for the middle group of trials (38-47) in the block. Each dot (n=16) is one confidence choice probability for a pair of stimulus strength and perceptual decision. Dot size is proportional to the total number of human confidence judgments for this pair of stimulus strength and perceptual decision. **(C)** The CNCB efficiency is decreasing over the block of trials. **(D)** The CNCB efficiency is approximately the square of the M-ratio. **(E)** The confidence noise is increasing over the block of trials. **(F)** The confidence boost is also increasing over the block of trials.

B. Faivre et al. (2018; Experiment 1)

The second example is a multi-sensory study by Faivre et al. ([2]; Experiment 1) where participants used a continuous scale to report their confidence. For each modality (audio, tactile, visual), each participant (N=15) first performed 400 trials on a perceptual decision followed by a confidence judgment on a continuous full scale from 0 to 1. Given that staircases were used to present the stimuli, we did not assume that there was a single stimulus strength, but instead split these stimulus strengths into 4 quantiles. For the purpose of estimating the goodness of fit, we divided the confidence ratings in 4 quartiles. We found that the CNCB model was a good fit for most of the participants in all three modalities. For the participant shown in **Figure 2A**, the analysis of the Deviance led to chi2(20) = 14.7, p = 0.794 for the auditory modality, chi2(20) = 20.1, p = 0.449 for the tactile modality, and chi2(20) = 20.2, p = 0.448 for the visual modality. This analysis reveals that for this participant the fit of the CNCB model was not significantly different from the best possible fit in all the modalities.

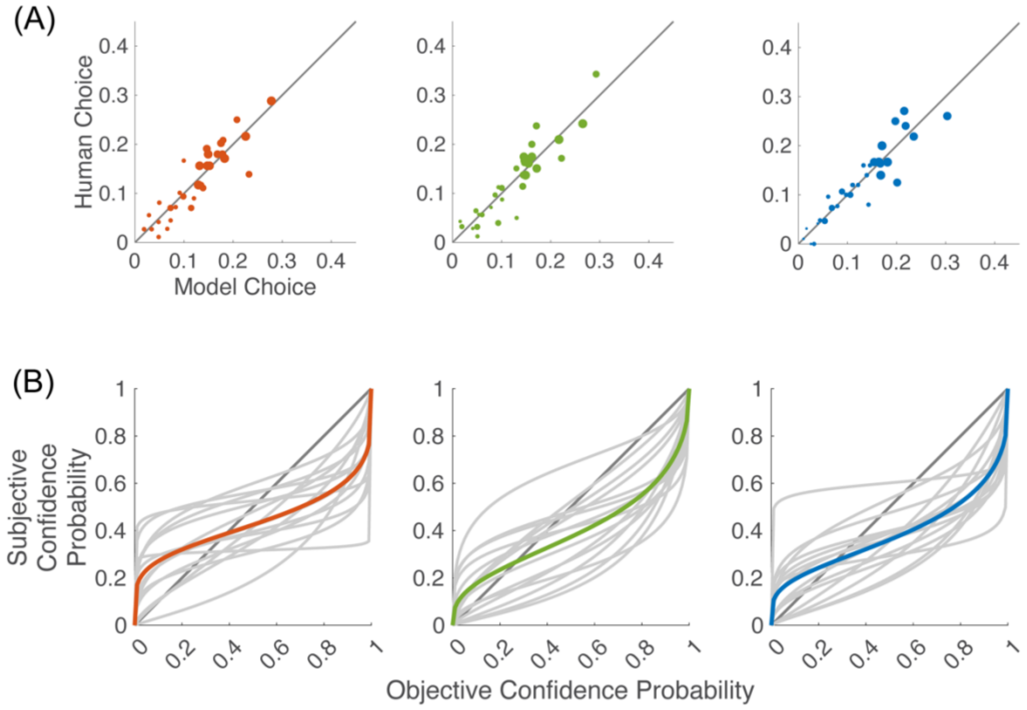

Figure 2. CNCB analysis of Faivre et al. [2], Experiment 1. **(A).** Goodness of fit of the CNCB model for a prototypical participant (#3). Each dot (n=32) is one confidence choice probability for a pair of stimulus strength and perceptual decision. Dot size is proportional to the total number of human confidence judgments for this pair of stimulus strength and perceptual decision. The three panels correspond to different modalities, audio (red), tactile (green), and visual (blue). **(B).** The plots show the non-linear mapping between objective and subjective confidence probabilities for the three modalities. Individual participants (N=15) are shown as grey lines, and the mean across participants as solid coloured lines.

We were particularly interested in the potential non-linear mapping between our model confidence probabilities and participants' reported confidence. We find that the γ parameter was less than 1 for most participants (**Figure 2B**). A value of γ less than 1 means that participants avoided to make extremely low or high confidence judgments. We might expect that a given participant is going to adopt the same non-linear mapping between objective and subjective confidence across all sensory modalities, and indeed, the γ parameters were similar across modalities: mean values 0.38 for audio,

0.58 for tactile, and 0.49 for visual. However, there were no significant correlations across participants apart from one that approached significance (Pearson correlations were R=0.074 (p=0.793) between audio and tactile, 0.206 (0.461) between audio and visual, and 0.513 (0.051) between tactile and visual). More work, with more participants, is needed to address the question of whether individual participants have a single non-linear probability transformation across tasks and across sensory modalities.

C. Shekhar & Rahnev (2021; Experiment 4)

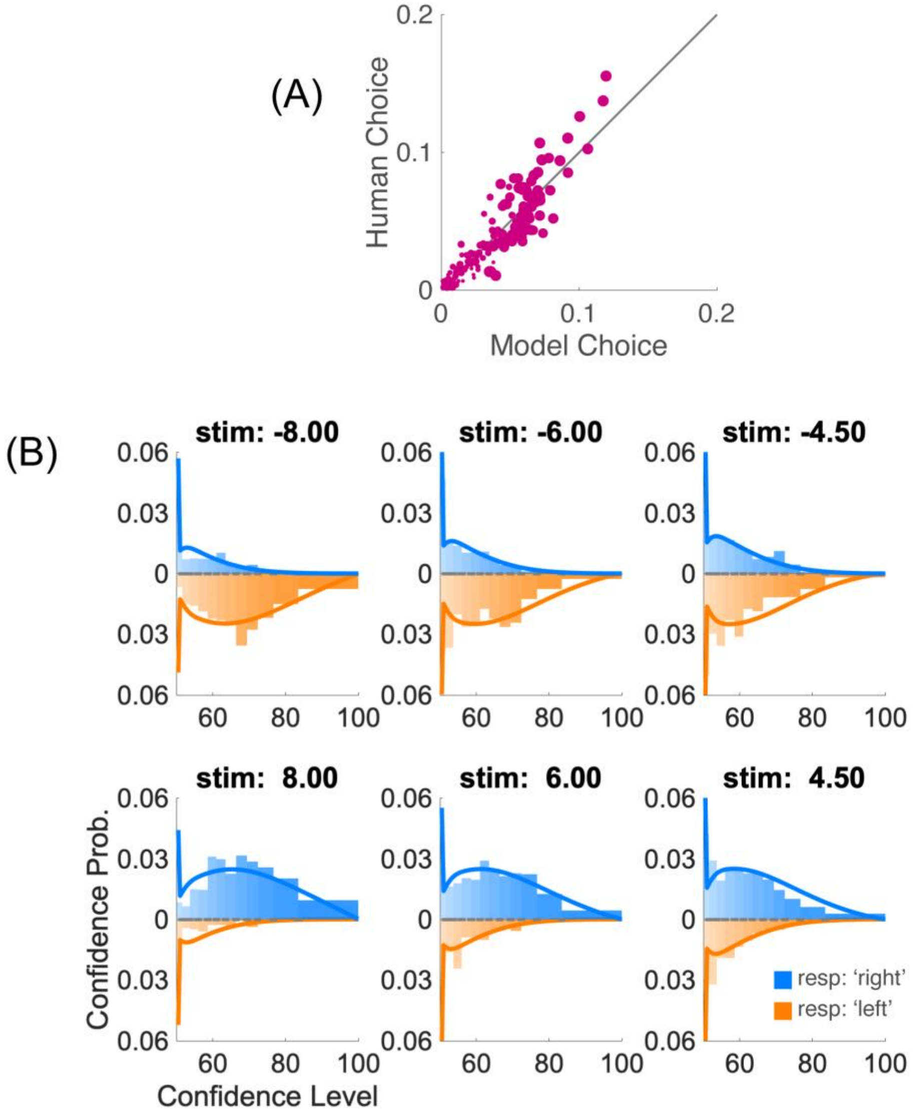

Figure 3. CNCB analysis of Shekhar & Rahnev [3], Experiment 4. **(A)** Goodness of fit to confidence ratings of participant #15 whose confidence judgments were quantized in 12 bins. Each dot (n=144) is one confidence choice probability for a pair of stimulus strength and perceptual decision. Dot size is proportional to the total number of human confidence judgments for this pair of stimulus strength and perceptual decision. **(B)** Comparison of confidence judgments of participants #15 shown as histograms and the CNCB model shown as continuous lines. Blue traces are for responses "right tilt" and orange traces (running downwards) are for responses "left tilt".

The last example is an experiment that was specifically designed to test the validity of different confidence models [3]; see also [4, 5]). In that experiment (Experiment 4 of Shekhar & Rahnev [3]), each of the 20 participants ran a large number of trials (2,800). The perceptual task was to discriminate the orientation (left or right tilt) of a Gabor patch whose contrast could take one of three values. These three stimulus strengths were interleaved within a block of trials. Simultaneously with the perceptual decision, participants had to report their confidence in this decision using a continuous half-scale from 50 to 100 (the scale was duplicated in mirror symmetry so that participants could use the left side to report a left tilt of the Gabor, and the right side for a right tilt).

We apply the CNCB model to each participant individually, but simultaneously to the three stimulus strengths and directly to the continuous confidence ratings (**Figure 3A**). To evaluate the goodness of fit, we quantize the continuous confidence ratings in 12 bins, and for a prototypical participant, the analysis of the Deviance showed that the likelihood of CNCB model was significantly different from the likelihood of the saturated model (chi2(128) = 667, $p < 0.001$; **Figure 3B**). We note that because a half-scale was used, the model presents a peak at the lower end of the confidence scale (rating of 50) that reflects when the model believed that the perceptual decision was wrong, whereas the human data do not present such a peak. Given that participants were requested to report simultaneously their perceptual decision and their confidence judgment, it is likely that if their confidence was less than 50%, they would have switched their perceptual response. If this was indeed the strategy used by the participants, a possibly better model would be obtained by folding the model confidence choices that are less than 50% over the actual range available to the participants (and switching the corresponding fraction of perceptual responses). However, such an analysis goes beyond the purpose of the current manuscript.

References

1. Bang JW, Shekhar M, Rahnev D. Sensory noise increases metacognitive efficiency. J Exp Psychol Gen. 2019;148(3):437-452. doi:10.1037/xge0000511

2. Faivre N, Filevich E, Solovey G, Kühn S, Blanke O. Behavioral, modeling, and electrophysiological evidence for supramodality in human metacognition. J Neurosci. 2018;38(2):263-277. doi:10.1523/JNEUROSCI.0322-17.2017

3. Shekhar M, Rahnev D. The nature of metacognitive inefficiency in perceptual decision making. Psychol Rev. 2021;128(1):45-70. doi:10.1037/rev0000249

4. Shekhar M, Rahnev D. How do humans give confidence? A comprehensive comparison of process models of perceptual metacognition. J Exp Psychol Gen. 2024;153(3):656-688. doi:10.1037/xge0001524

5. Guggenmos M. Reverse engineering of metacognition. Elife. 2022;11:e75420. doi:10.7554/eLife.75420
