# Supplementary material for: The confidence-noise confidence-boost (CNCB) model of confidence rating data
Source: PLoS Comput Biol. 2025 Apr 21;21(4):e1012451. doi: 10.1371/journal.pcbi.1012451 (PMC12043244; doi:10.1371/journal.pcbi.1012451)
Supplement: Attachment — Submitted filename: response_letter_241129.pdf [file pcbi.1012451.s003.pdf]

Response to the reviewers

Dear editor,

We would like to thank you and the reviewers for the detailed comments and for offering us the possibility to revise our manuscript. Here is a summary of the main changes:

- we have added a measure of the quality of the parameter recovery in terms of a computation of AUROC,
- we have added an analysis of model recovery,
- we have added simulations of the effects of sensory noise,
- we have added simulations of the effects of confidence bias,
- we have generalized the comparison of the CNCB efficiency and the M-ratio over a wider range of model parameters,
- we have added practical recommendations to recover the confidence noise and boost parameters of the CNCB model,
- we have updated the description of the likelihood function in the section "Materials and Methods", subsection "Fitting procedure", and
- we have moved the Appendix to a separate "Supporting Information" file (as requested).

Overall, we believe that we have addressed all the reviewers' comments, and the revised manuscript has clearly improved in clarity.

Reviewers' comments are reproduced below in black, and our replies in blue. Changes in the revised manuscript are also shown in red.

On behalf of the authors,
Pascal Mamassian

--

Dear Dr. Mamassian,

Thank you for submitting your manuscript to PLOS Computational Biology. After careful consideration, we feel that it has merit but does not fully meet PLOS Computational Biology's publication criteria as it currently stands. Therefore, we invite you to submit a revised version of the manuscript that addresses the points raised during the review process.

[…]

We look forward to receiving your revised manuscript.

Kind regards,

Christoph Strauch

Academic Editor
PLOS Computational Biology

Marieke van Vugt
Section Editor
PLOS Computational Biology

Feilim Mac Gabhann
Editor-in-Chief
PLOS Computational Biology

Jason Papin
Editor-in-Chief
PLOS Computational Biology

Journal Requirements:

Additional Editor Comments (if provided):

The reviewers (and we) have found the manuscript to be of interest. Yet, the expert reviews identify a list of issues that would need being addressed before reaching a final decision for the manuscript. Besides the comments given below, one reviewer further noted that they could not find the scripts implementing the simulations, please double-check/clarify in a revision.

[Note: HTML markup is below. Please do not edit.]

We have added the scripts for the simulations, as well as the data files for the simulations themselves. They are available here:

Mamassian, P. (2025). Simulations of the CNCB model for the analysis of confidence ratings data (0.1) [Data set]. Zenodo. https://doi.org/10.5281/zenodo.14632514

Reviewers' comments:

Reviewer's Responses to Questions
Comments to the Authors:
Please note here if the review is uploaded as an attachment.
Reviewer #1: This paper presents a significant advance over the standard modelling tools available to infer metacognitive sensitivity from confidence ratings. The authors build on their recent Psych Review paper, which devised a similar model for 2IFC confidence judgments, to show how fitting this model to rating data (the type 2 ROC) can recover two distinct parameters, confidence noise and confidence boost, that map onto distinct processes that have been posited in the confidence literature (eg metacognitive noise vs. additional information from post-decisional processing and other sources).

This will be a valuable tool and model for psychologists and neuroscientists interested in studying the underpinnings of confidence formation and metacognition. Accompanying the paper is a toolbox that should help this method gain widespread usage. The extension to model continuous ratings, and nonlinear mappings between subjective confidence and objective probabilities, is also impressive.

My main comment is about the extent to which the model and parameters can be effectively recovered – as establishing this is key to its advance beyond a single-parameter model like meta-d, and central to its practical usefulness. This comment can be split into a few different queries:

1) More general comment: in the figures, the focus is on parameter recovery, but not model recovery. The data are simulated from a two parameter (boost and noise) model, and these two parameters are recovered reasonably well (see next point). But this does not tell us whether there is the potential for confusion between model architectures – or whether the more complex model is justified in the first place. In other words, if you simulate data from a one-parameter model (only confidence noise, no boost – similar to classical SDT) then can this model be recovered (in comparison to the more complex two-parameter case)? And perhaps more importantly, if you simulate data from the new two-parameter model, can you show that this is preferred to a one-parameter noise-only model (over a reasonable range of parameter values)?

We thank the reviewer for pointing out this missing analysis from our manuscript. Our previous analysis of the recovery of the confidence boost parameter was a partial answer to that request, since the one-parameter model is a two-parameter model with a confidence boost equal to 0. We have now performed a proper model recovery analysis and found that the one-parameter model was systematically recovered (99% of the times). Regarding the two-parameter model, it was recovered on average 74% of the times, and not too surprisingly, the errors occurred when the confidence boost parameter was small.

We have added a new section called “Model recovery” in the manuscript to detail this model recovery analysis, including a new figure (Figure 7).

Model recovery

The previous two sections have shown that the confidence noise and confidence boost parameters could be disentangled when the experiment involves more than two confidence levels or more than two stimulus strengths. This suggests that models with or without the confidence boost parameter could be distinguished. We now address this question more directly by performing a model comparison analysis.

We have simulated data from two types of models. Model 1 does not contain a confidence boost parameter (it is equivalent to setting this parameter to zero), and Model 2 does contain a confidence boost parameter that is drawn randomly on its domain (from 0 to 1) across simulations. In both cases, we used six stimulus strengths and 4 confidence levels. We also randomized sensory noise, sensory

criterion, confidence noise, and confidence bias (see section Materials and Methods for details). We then fitted these simulated datasets by the two types of models (without and with a confidence boost parameter) and check which model best fit the data. We used likelihood ratio tests for nested models [19] to test whether Model 2 is better than Model 1. We computed the test statistic $\lambda_{\text{LR}} = -2(\lambda_1 - \lambda_2)$, where λ_1 (resp. λ_2) is the log likelihood of Model 1 (resp. Model 2). If Model 1 is correct, then this test statistic is asymptotically distributed as a χ_1^2 random variable (the degrees of freedom is 1 because the two models differ by only one parameter).

The simulations indicate that the two models are reasonably well recovered over a reasonable range of other parameters' values (**Figure 7A**). When Model 1 was simulated, it was recovered 99% of the times, and when Model 2 was simulated, it was recovered 76% of the times, thus resulting in a discrimination sensitivity of the two models of $d' = 3.24$, with a bias in favour of Model 1 (discrimination criterion: $c = 0.92$). The bias is expected because when Model 2 is simulated with a small confidence boost, this model is not distinguishable from Model 1 (**Figure 7C**, right panel). When Model 2 is simulated with a large confidence noise, there is also greater confusion with Model 1 (**Figure 7C**, left panel). When Model 1 is simulated, recovery of that model is very good, and estimated confidence noise and boost are also very good (**Figure 7B**).

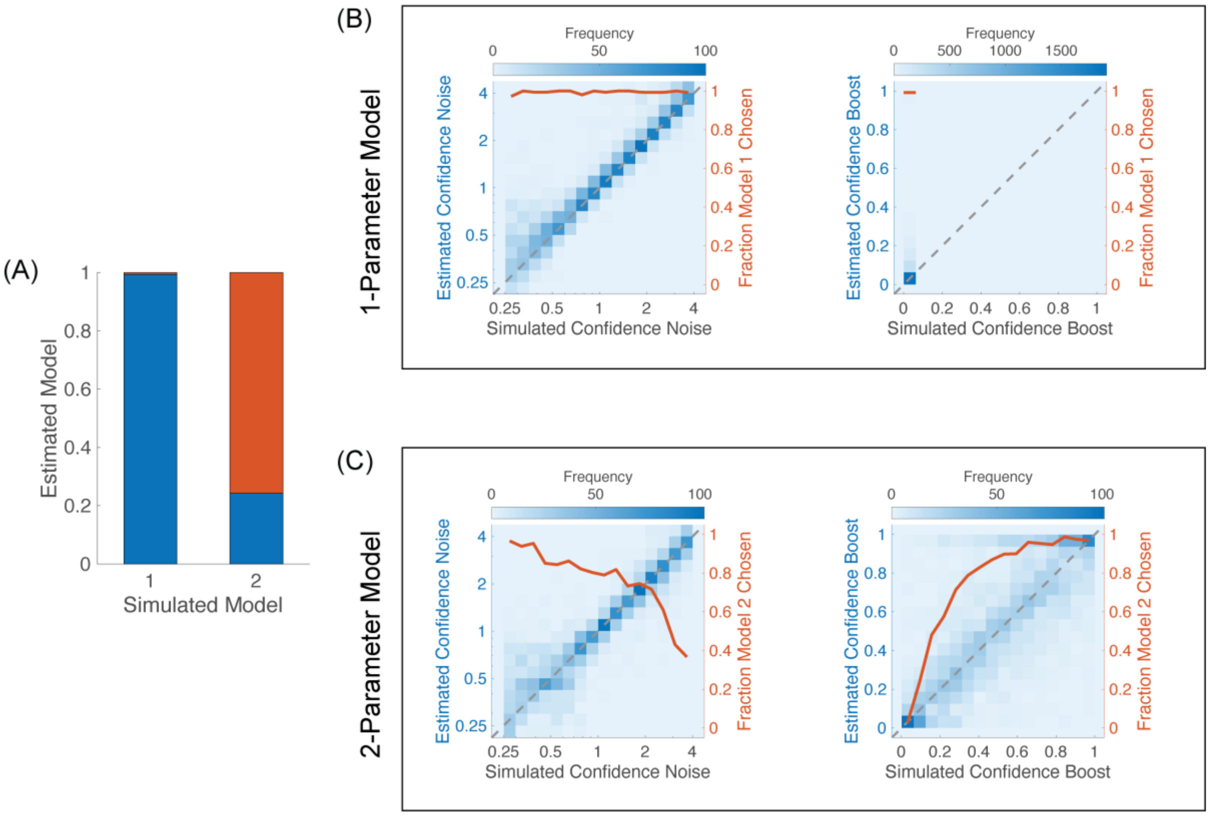

Figure 7. Model recovery. (A) Fraction of estimated models for each simulated model. Model 1 does not have a confidence boost parameter and Model 2 does. Blue bars correspond to fractions of estimated Model 1, and red bars Model 2. (B) Simulations of Model 1. Heatmaps are parameters' estimates from Model 2, with saturation showing the

frequency of estimated parameter over 2,500 simulations. Left panel, left axis: estimated confidence noise as a function of simulated confidence noise. The diagonal dashed line shows perfect performance. The axis on the right and the red curve show the fraction of times Model 1 was favoured over Model 2 as a function of confidence noise. Right panel: same analysis as that in the left panel, but for confidence boost instead of confidence noise. (C) Simulations of Model 2. Left and right panels show the effects of simulated confidence noise and confidence boost, respectively, on the estimated confidence noise and confidence boost, and on the fraction of times Model 2 was favoured over Model 1.

2) What counts as “good enough” parameter recovery here? It would be good to have some more quantitative metric of this. Eyeballing the plots, it looks like noise and efficiency are cleanly recovered in simulation, but that confidence boost is harder to pin down (eg Figs 4C, 6C), and also trades off against the noise parameter (which is another reason why quantifying confusability between single- and two-parameter architectures seems important). In Fig 7I, the error variance in boost recovery only comes down to acceptable levels in > 10,000 trials – which casts doubt on whether we should trust estimates obtained in fits to real datasets (eg as reported in the Appendix).

We thank the reviewer for requesting a measure of goodness of parameter recovery. The current practice in the literature is to look at the correlation between estimated and simulated parameters. In our simulations, these correlations are very high, but they are also very crude measures of parameter recovery. Therefore, we followed a different approach. We computed the discriminability of the distributions of estimated parameters for two reasonably distinct parameters while keeping all the other parameters of the model constant. We plotted this discriminability (in the form of the area under the ROC curve, AUROC) whenever it made sense. For instance, the AUROC for discriminating confidence boost was above a set-threshold of 0.75 when the number of trials was larger than 2,000.

We have added a description of this analysis the first time we performed it (lines 265-268).

To quantify the reliability of the efficiency measure, we computed the discriminability between two conditions (here the conditions where confidence noise and boost are $(0.5, 0.2)$, $(0.5, 0.8)$). The discriminability measure is the area under the ROC to separate the distributions of efficiencies obtained from the simulations.

3) Following on from the previous point – in Figure 12D, inferred confidence boost is binary, being either 1 or 0 on different (sequential!) trials of the task. This seems implausible and also suggests the limited reliability of this parameter in real datasets.

Following the previous point, we have binned consecutive trials to have enough power to estimate the confidence boost parameter. See the new Figure 1 in the Supporting Information in replacement of the original Figure 12.

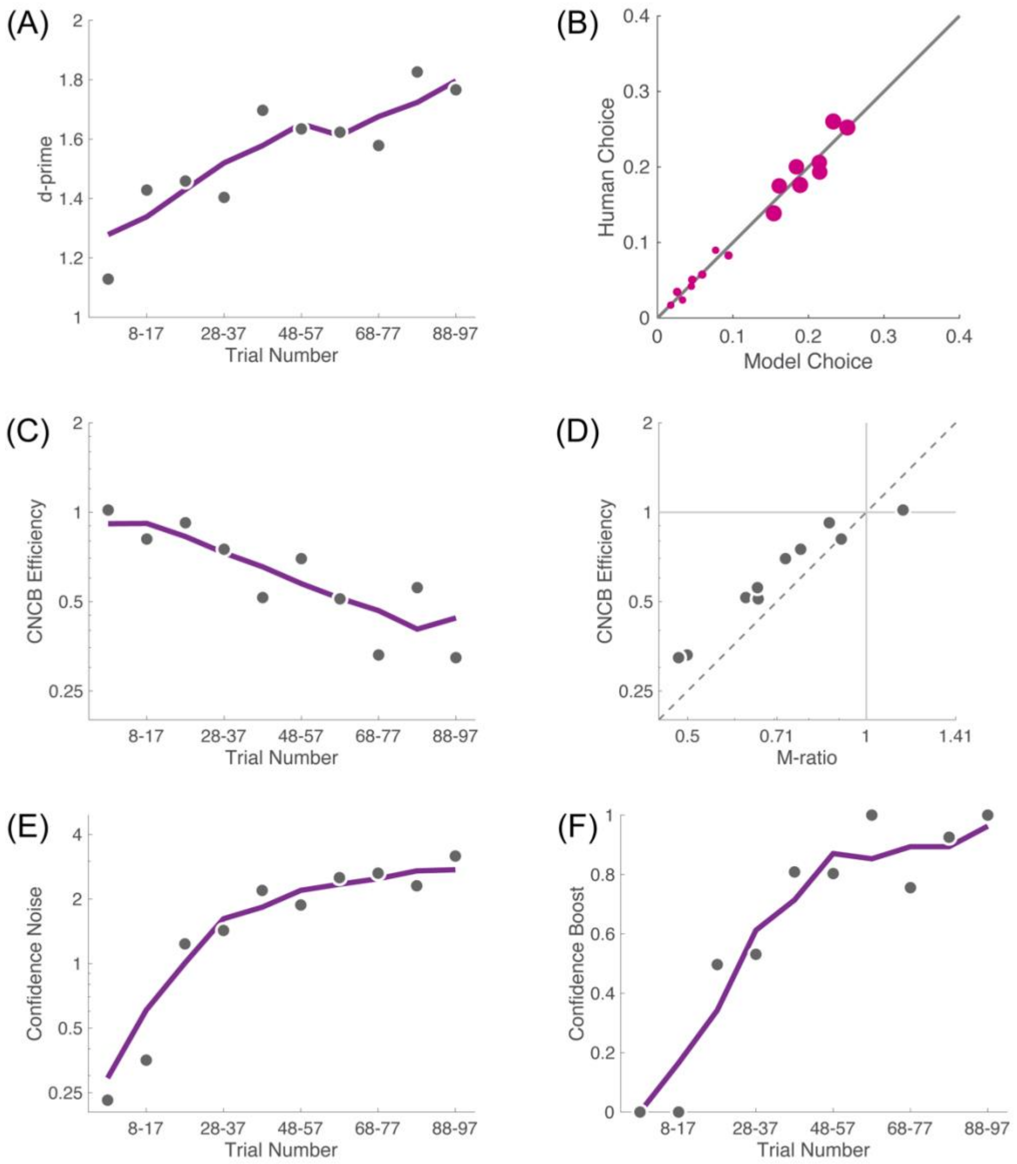

Figure 1. CNCB analysis of Bang et al. [1], Experiment 2, Task 1 (coarse discrimination). **(A)** Perceptual sensitivity (d') is increasing over the block of trials. Each dot corresponds to the sensitivity for a group of 10 trials (except the first bin that contains only 7 trials) computed by pooling all observers. The solid line is the running average over 3 consecutive groups of trials. **(B)** Goodness of fit of the CNCB model for the middle group of trials (38-47) in the block. Each dot (n=16) is one confidence choice probability for a pair of stimulus strength and perceptual decision. Dot size is proportional to the total number of human confidence judgments for this pair of stimulus strength and perceptual decision. **(C)** The CNCB efficiency is decreasing over the block of trials. **(D)** The CNCB efficiency is approximately the square of the M-ratio. **(E)** The confidence noise is

increasing over the block of trials. **(F)** The confidence boost is also increasing over the block of trials.

4) On p. 9, it's asserted that having more points on the type 2 ROC helps disentangle the confidence noise and confidence boost parameters. But why? Doesn't figure 2 show that there are theoretical type 2 ROCs that are equivalent in boost and noise? How does having more points along these curves help with this?

The reviewer is correct, Figure 2 shows that an increase of confidence boost can be traded off by an increase of the confidence noise. This property is what helps us define the equivalent confidence noise, and then the CNCB efficiency measure. However, this property only holds strictly when there are only two confidence levels.

An intuition for why confidence noise and confidence boost can be disentangled when there are more than two confidence levels is shown on the Type 2 ROC in Figure 2A. In that figure, the blue curve shows the ROC for the pair of confidence noise and boost equal to (0.5, 0.2). However, because there are only two confidence levels, the ROC is reduced to a single point shown in blue. When we set the confidence boost to 1, we can always find a confidence noise so that the new Type 2 ROC (shown in dashed green) goes through this single point of the ROC. But we see that the two ROC curves have only that single point in common. Outside of this intersection, the curves will slightly differ, as illustrated in Figure 2A. When we add more confidence levels, we can no longer find new pairs of confidence noise and confidence boost that bring the new ROC curve in contact simultaneously with all the points of the ROC.

We have added some text in the manuscript to clarify this point (lines 240-243):

Indeed, whereas a single point in the Type 2 ROC space was compatible with many Type 2 ROC curves intersecting on this single point (and thus with many pairs of confidence noise and boost), specifying more than one point sets stronger constraints by which a single curve, and thus a single pair of confidence noise and boost parameters, can be identified.

Minor points:

- P. 3, bottom, I wasn't convinced that you need to know the generative process to have a "proper measure of efficiency". By analogy – d' is a proper measure of sensitivity, but is compatible with a number of different process models of perception or memory etc.

This comment was to contrast the approach developed here with the meta-d' measure that is purely a fitting approach and for which there are no obvious generative process. Although we agree that the perceptual sensitivity measure d' is most often used without consideration for the underlying process, its theoretical construction d' is based on a

generative process model whereby noisy sensory evidence is sampled from an uncertainty distribution of the stimulus in some internal representational space.

We replaced this sentence by the following (lines 73-74):

As a consequence, values of M-ratio cannot be easily mapped onto specific internal parameters and in particular values greater than 1 are hard to interpret.

- Eq. 3, is this a new draw of epsilon_s? Or is it the same sample used in Eq. 1? (I'm presuming the latter, but useful to make this explicit)

Thank you for this comment that helped us clarify this point. It is indeed the same \epilson_s in both Equations 1 and 3. This has been clarified (see line 134):

In this equation, the sensory noise ϵ_s is the same as in Eq. 1.

- Eq. 4, for a moment I thought that Dw was a new variable until I realised it was the product of D and w. Perhaps make this clearer using product notation.

Thank you for this comment. We have implemented this change.

$$\begin{cases} C = \text{High} \quad \text{if} \quad D.w > b\,, \\ C = \text{Low} \quad \text{if} \quad D.w \leq b \end{cases}, \qquad \text{(Eq. 1)}$$

- P. 7, what does "secondary information" mean here? (line 180)

By "secondary information", we mean confidence evidence that is distinct from the evidence obtained from the sensory stream of information processing. To clarify, we now write that this is an observer who has "full access to the stimulus" (line 186).

- Line 488, "confidential" – I'm not sure this is the right word here. Perhaps "less influential"?

Thank you for this suggestion. We have replaced "confidential" by "less influential" (line 603).

- I felt that the introduction omits consideration of recent attempts to innovate along the same lines as the CNBC model. Most notably, Boundy-Singer et al. Nat Hum Behav develop a model of metacognitive efficiency that can be fit across different stimulus strengths, and offers a process level interpretation of deficits in metacognition as failures to appropriately track sensory noise (meta-uncertainty). This model is cited in the discussion, but it was not clear how the authors see their efforts in relation to this. For instance, when reading the sentence "…but with no clear answer to the question of how to formally define and

measure the efficiency with which human observers produce confidence judgments", I found myself thinking "but what about Boundy-Singer and other attempts to do just this?". Tackling this head on in the introduction would seem useful to ensure that the contribution of the paper is properly situated. How does the CNBC model go beyond this, or how does it take a different path?

Our model shows some similarities to the model of Boundy-Singer et al. in that confidence evidence is normalized by an estimate of sensory noise, but also some differences. The main difference is that our estimate of sensory noise can be biased but there is no uncertainty attached to it. In contrast, we introduce confidence noise as additive noise like more classical models of confidence, and we introduce confidence boost, which has no equivalent in the model of Boundy-Singer et al.

We have added a proper reference to the paper of Boundy-Singer et al. in the introduction, highlighting this last element (lines 91-93):

We call this additional information the confidence boost, and note that this component is specific to our approach compared to other work [14].

- Again on framing – I really liked the extension of the model to incorporate nonlinearities in probability functions. It's very nice that these parameters can be recovered independently of sensitivity, as it starts to provide a model-based formulation of metacognitive biases / overconfidence. It also integrates more closely the JDM / probability judgment and metacognition literatures. Perhaps this ambition can be flagged more clearly to the reader in the introduction.

Thank you for this suggestion. We have updated the introduction to reflect this ambition. Our last paragraph in the introduction now ends with these sentences (lines 98-102):

As we demonstrate below, this framework is applicable to both discrete confidence ratings and continuous estimations on a (possibly distorted) probability scale. By doing so, our approach opens possibilities to develop model-based formulations of overconfidence, and to bridge a gap between metacognition research and the literature on judgment and decision making.

- I wasn't convinced that the ability to accommodate more than one stimulus strength is a major advance over meta-d (p. 13). It would be straightforward to fit a constant ratio to multiple meta-d/d points at different d' values – and this was indeed done in some of the earlier work by Maniscalco & Lau.

While it is indeed possible to fit a constant M-ratio for different d' values, we think this is dangerous. This procedure is similar to trying to compute multiple d' values from a psychometric function that has more than 2 stimulus strengths. The problem is that when there are more than 2 possible stimuli, the false alarm rate is difficult to compute. Let us take an example with 6 stimulus strengths; in this case, the false alarm rate would be

P(response is A | stimulus is not A), but there are 5 possible stimuli that contribute to the conditional term, and it is complex to combine all these scenarios.

We have clarified our position in the text (lines 355-357):

Our approach, by contrast, naturally accommodates multiple difficulty levels by estimating a single sensory noise, and thus takes advantage of such designs to provide more refined estimates of subcomponents of the metacognitive process.

Signed: Steve Fleming

--

Reviewer #2: The authors present a generative model of confidence judgments, the confidence noise and confidence boost (CNCB) model. The model is an alternative to approaches based on meta-d' and assumes that, in addition to the sensory signal, confidence judgments can be further influenced by (1) information beyond the sensory signal can influence confidence, and (2) the confidence signal can be perturbed by its own source of noise. These two additional points of influence are represented as distinct model parameters, confidence boost (alpha) and confidence noise (sigma_c), respectively. Part of the motivation for developing the CNCB model is that it has a wider range of applications compared to meta-d' in that CNCB can be used with multiple difficulty levels as opposed to just one.
An issue explored with the new model concerns tradeoffs between the confidence boost and confidence noise parameters. The authors show that the two parameters can be estimated well when there are more than two levels of confidence available for report and/or levels of stimulus strength (or task difficulty).
Parameter recovery analyses showed that the confidence boost and confidence noise parameters could be recovered well across a wide range of sample sizes and that the model could capture confidence efficiency across a range of circumstances. Further exploration shows a one-to-one correspondence between confidence efficiency as measured by the CNCB model and the m-ratio derived from the meta-d' framework.

My evaluation of this manuscript is quite positive. The focus here is on the properties of the CNCB model and the authors have showcased how it is a potentially useful alternative to meta-d'. My main concerns are about the confidence boost parameter (alpha) and the relatively limited exploration of the CNCB's parameter space. I discuss these two points below and include a third more minor point of confusion in the exposition.

We thank the reviewer for their thorough and positive evaluation of our work.

1 – The effect of the confidence boost parameter is to reduce (or, when alpha = 1, entirely bypass) the influence of sensory noise on confidence (Equation 3). I think this is an interesting idea, but I am struggling to wrap my head around how to interpret this

mechanistically (and if it is even appropriate to do so—it might not be and that's fine). Is the parameter intended as something of a catch-all that captures all of the outside influences on the sensory signal for the purposes of making confidence judgments without committing to identifying exactly what they are? My sense is that the CNCB's main use is as a vehicle for measuring confidence and confidence efficiency and so perhaps there is no need to think mechanistically about this parameter.

The reviewer is correct, the confidence boost parameter captures all the sources of confidence that are not extracted from the sensory decision process. While it would be nice to be able to separate these different sources in the future, they were combined here for simplicity. This point was already discussed in our previous work where we have introduced the confidence boost parameter (Mamassian & de Gardelle, 2022, Psych Rev).

That said, even if confidence boost is intended to summarize "outside" effects on confidence, it would be helpful if the authors could describe experimental manipulations that might be expected to influence this parameter (the same would apply to confidence noise). For example, are there task conditions that would be expected to systematically influence confidence boost independent of confidence noise and vice-versa? The demonstrations of separable parameter estimation are important but a big question to me is whether the parameters can be independently manipulated experimentally.

We thank the reviewer for highlighting this important point. At this stage, we can only speculate about experimental manipulations that should specifically influence the confidence boost parameter without influencing any of the other parameters. One such manipulation may be the delay between the perceptual decision and confidence judgment. Increasing this delay should increase confidence boost because participants may have more time to reflect back on the stimulus. We found some evidence for this, but only for high-level tasks (Balsdon et al., 2024).

We have updated the text by mentioning this result in our introduction (lines 93-94):

It is interesting to think how confidence boost could be manipulated experimentally, for instance by varying the delay between Type 1 and Type 2 responses [15].

2 – I think the demonstrations that multiple levels of confidence and stimulus strength (pp. 8-13) can help disentangle confidence boost from confidence noise are extremely interesting, but I was unsure what they added beyond the parameter recovery results reported on pp. 13-14. If I understand what was done correctly, aren't these essentially small-scale parameter recovery studies? My sense is that a better approach might be to investigate how well parameters recover when generating values of confidence boost and noise are both randomly sampled as a function of the number of confidence levels/stimulus strengths. I think that doing things this way will provide a clearer picture of what is sketched in the current sections but it would also allow clearer examination of correlations across these parameters for fits to data.

The reviewer is correct in stating that our simulations are slices of the parameters space where we focus on one parameter at the time and keep all the other parameters constant. We believe that this is a more informative approach than just randomly sampling multiple parameters and looking at the correlations between simulated and recovered parameters. Our approach reveals some biases for specific combinations of parameters that would otherwise be buried in correlation plots, for instance the under-estimation of high values of confidence boost but only when confidence noise is low (see Figure 8F). These biases would deserve to be studied further in future research to understand why they occur.

Even though we believe our analysis of slices of the parameters space is more information, we have performed some simulations along the lines suggested by the reviewer. We present the results of these simulations in the new Figure 7. In these simulations, confidence noise and boost were randomly sampled, and we performed parameter recovery when there were 6 stimulus strengths and 4 confidence levels. In this case, parameter recovery was excellent (see our response above to the 1st point of Reviewer 1).

3 – Page 7 Lines 173-182 – I had trouble following the argument in this passage. In describing the equivalence of the blue dot (simulated data) in Figure 2A with the green dot (ideal confidence observer) in Figure 2B, is this a general statement that the ideal confidence observer can produce equivalent performance to (any?) human/simulated observer, or is the argument about this one specific case?
In the end, I understood this passage to be illustrating a potential problem with estimating confidence boost and confidence noise separately (hence the demonstrations on pp. 8-13), but this was not clear to me upon first reading.

We apologize for the lack of clarity in this important part of the manuscript. The green dot is not representing the ideal confidence observer, it is the equivalent confidence noise of the blue dot. The ideal confidence observer is shown as the black curve. The argument is that any blue dot (any pair of confidence noise and boost) will have a corresponding green dot (an equivalent confidence noise when the confidence boost is set to 1). Since this argument is generic, it also applies to the ideal confidence observer that, by definition has confidence noise and boost equal to 0, and the equivalent confidence noise for the ideal confidence observer is τ_{ideal}.

We have clarified this point in the manuscript (lines 183-185):

In fact, all these pairs that are compatible with this Type 2 ROC point can be represented as a continuous set (see Figure 2B), and this set can be fully characterized by taking the most extreme pair in the set, that is the one for which confidence boost is 1 (green dot in Figure 2B).

Line 117 – Typo, "…what the observer report[s] as their confidence…"

Fixed, thank you!

--

Reviewer #3: In this paper Mamassian & de Gardelle present their confidence noise confidence boost (CNCB) model for analysing confidence data. I think elements of the model are interesting, but there are aspects of the model and it's behaviour I'm left unclear on.

We thank the reviewer for their thorough reading of our manuscript.

Major comments:

1. There is something I don't understand in the model, specifically, in equation 3 (the specification of confidence evidence w). In the way that w is formulated, and as far as I can see, if a = 1 then the model says confidence has direct access to the stimulus evidence. This assumption cannot possibly hold. I would understand if confidence boost weighted the sensory evidence, i.e. w = (1-a)*(mu_s + e_s)…, so that when confidence boost is 1 the participant is estimating perceptual confidence blind to the sensory evidence. However, this doesn't seem the case. I appreciate that with the internal noise parameter w could be reformulated to get around the problem, but it wouldn't change the conceptual issue.

The reviewer is correct in thinking that $\alpha = 1$ is a limiting case where confidence has direct access to the stimulus. Like any limiting case, even though one cannot reach this scenario in practice (it is implausible that the observer has access to 100% of the information contained in the stimulus), one could get very close to it, and so it is important to include this limiting case in the model.

The reviewer is suggesting a model where the confidence boost is modulating the sensory evidence. This model weakens the influence of the sensory evidence when α is large, but does not provide any new confidence evidence from the stimulus above and beyond what has been computed from the perceptual decision. Instead, our Equation 3 in the manuscript can be seen as a weighted sum of two sources of evidence, one coming from the perceptual decision and the other from any other source. In our previous work (Mamassian & de Gardelle, 2022), we called this latter the "super-ideal confidence observer" (see Equation 5 in that previous paper).

We have tried to clarify this point in the manuscript (lines 125-130):

In our framework, this confidence evidence is based on the initial sensory evidence s, with weight $(1-\alpha)$, and on additional information from the stimulus μ_s with weight α. We call α the boost parameter, which varies between 0 and 1. A value of 0 corresponds to a direct link between sensory evidence and confidence evidence, whereas a value of 1 corresponds to a perfect access to the stimulus, bypassing the impact of sensory noise on confidence judgments (we previously referred to this extreme case as the super-ideal confidence observer [4]).

2. A lot of the CNCB model is very similar to meta-d, to the extent that I'm struggling to see

why and how they are not mathematically equivalent (or, indeed, why CNCB is theory driven while m-ratio is not). The paper (including in the introduction and discussion) would benefit from more discussion and exploration of how they differ (and when they're the same), their respective costs and benefits, etc.

While CNCB efficiency is very close to the square of the M-ratio (see new Figure 12), there are some deviations between the two measures, in particular when there is a perceptual response bias (see Figure 10). Overall, the CNCB model shares with meta-d' the way confidence noise decreases confidence sensitivity. However, the CNCB model goes beyond meta-d' in offering a reason why meta-d' can sometimes be larger than d' (precisely thanks to the α parameter discussed in the previous point of the reviewer). The framework behind meta-d' is not detailing why it can sometimes be larger than d', it is just presenting a fitting procedure. We have clarified this main difference between the two approaches in the introduction (lines 90-93):

Second, observers can use information for their confidence judgment that was not used for the perceptual decision. We call this additional information the confidence boost, and note that this component is specific to our approach compared to other work [14].

3. There were also some simple demonstrations of the model's behaviour that were missing. For example, what is predicted (in terms of efficiency, mostly) when type 1 sensitivity and bias are manipulated?

We thank the reviewer for emphasizing the influence of sensory performance on confidence judgments. The effects of Type 1 parameters are now regrouped in a new section "Influence of sensory parameters". There were already simulations of Type 1 bias (Figure 9 of the original manuscript, now Figure 10) and we used these simulations to show that the CNCB efficiency was less biased than the M-ratio. We have now added simulations of Type 1 sensitivity (see lines 440-446 and new Figure 9). The CNCB model is very robust against variations of sensory noise.

Influence of sensory parameters

In this section, we investigate how the Type 1 sensitivity and bias affects the recovery of the Type 2 parameters. To manipulate Type 1 sensitivity, we varied the sensory noise over a range that led to a probability of correct perceptual decisions over $[0.55, 0.95]$ if the sensory criterion was optimal. We can see that both the M-ratio and our CNCB efficiency measures are less precise and less accurate when the probability of being correct is above 0.85 or below 0.6 (**Figure 9A**.**B**). Likewise, the recovery of the confidence noise and boost parameters are better when the probability of being correct is below 0.90 or above 0.6 (**Figure 9C**.**D**).

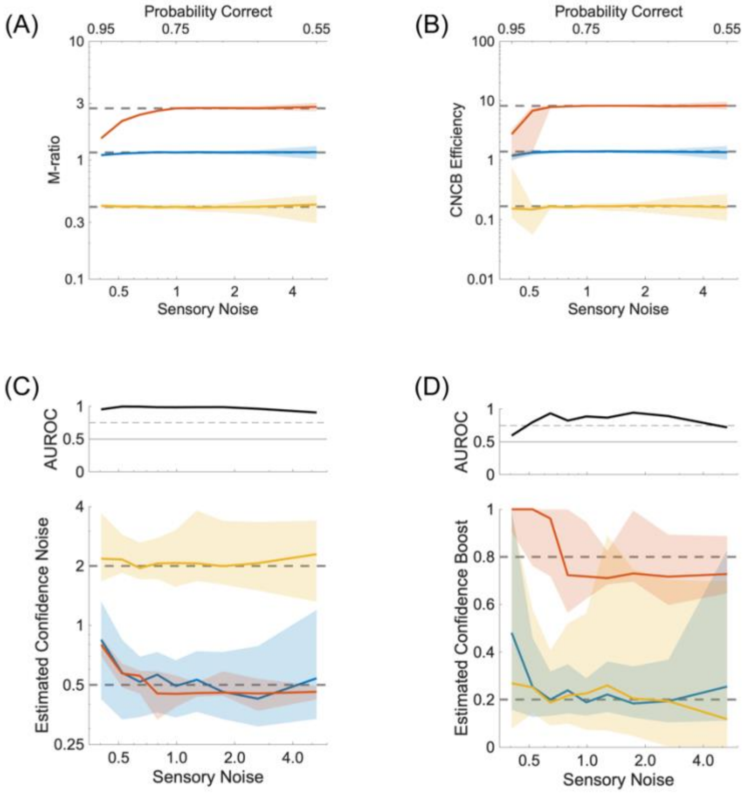

Figure 9. Effect of sensory sensitivity. **(A)** M-ratio as a function of different values of sensory noise. The colours represent three conditions for the confidence noise and confidence boost parameters (σ_c, α), namely $(0.5, 0.2)$ in blue, $(0.5, 0.8)$ in red, and $(2.0, 0.2)$ in yellow. Continuous lines and shaded areas show the medians and interquartile ranges, respectively, over 100 repeated simulations. The dashed lines correspond to the median M-ratio obtained for the sensory noise that has been used in the other simulations $(\sigma_s = 1.0)$. **(B)** CNCB efficiency estimates for the same conditions as in (A). **(C)** Estimated confidence noise. Upper panel: Discriminability of the blue and yellow simulations. **(D)** Estimated confidence boost. Upper panel: Discriminability of the blue and red simulations. Other parameters are listed in the Methods section.

Do you get the same efficiencies predicted when confidence bias is present (i.e. when the condition that confidence bins are used equally often is relaxed)? Some of this information was presented indirectly, late in the Results when comparing to m-ratio or applying the measures to continuous confidence data, but I think an explicit discussion of how the measures change with typical manipulations seen in experiments would be helpful.

The reviewer is correct in that we had only considered scenarios where the confidence judgments were equally spread across all confidence bins. We now present simulations

when there are over- or under-confidence judgments. These results are presented in the new Figure 11. The CNCB model is very robust against confidence biases (lines 490-499).

Influence of confidence bias

So far, we have assumed that the confidence judgments were distributed uniformly across all confidence levels. This helped minimize the possibility that some confidence levels were never visited for some combinations of stimulus strengths and perceptual decisions. We relax here this constraint to look at the effect of confidence biases on the recovery of the confidence parameters. To simulate the effect of a confidence bias, we replaced the uniform distribution of confidence levels by a triangular distribution, where the number of confidence judgments increases (or decreases) linearly across the confidence levels. The parameter δ describes the ratio of confidence judgments between the top and bottom levels. If it is larger than 1, there will be a dominance of high confidence values. The simulations shown in **Figure 11** indicate that the recovery of confidence noise and boost is quite robust over a large range of confidence biases.

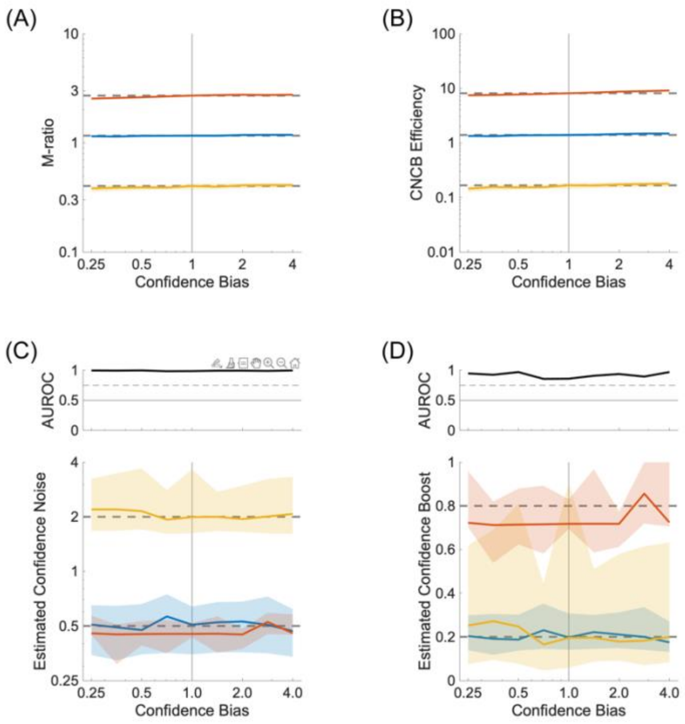

Figure 11. Effect of confidence bias. **(A)** M-ratio as a function of different values of confidence bias. The colours represent three conditions for the confidence noise and confidence boost parameters (σ_c, α), namely $(0.5, 0.2)$ in blue, $(0.5, 0.8)$ in red, and $(2.0, 0.2)$ in yellow. Continuous lines and shaded areas show the medians and interquartile ranges, respectively, over 100 repeated simulations. The dashed lines

correspond to the median M-ratio obtained for a uniform distribution of confidence judgments across all levels, presumably leading to the least bias in M-ratio estimate. **(B)** CNCB efficiency estimates for the same conditions as in (A). **(C)** Estimated confidence noise. Upper panel: Discriminability of the blue and yellow simulations. **(D)** Estimated confidence boost. Upper panel: Discriminability of the blue and red simulations. Other parameters are listed in the Methods section.

Similarly, it would be helpful to see how confidence noise, boost, and CNCB efficiency vary with factors like type 1 threshold and sensitivity on real data, and to compare CNCB efficiency against m-ratio with real data as well.

We thank the reviewer for this suggestion. We had presented in the Appendix 3 examples of how well the CNCB model fits real data. The Appendix has now been shifted to a separate “Supporting Information” file. In that file, we have now added a plot of the relationship between CNCB efficiency and M-ratio for one of the examples in Figure 1D.

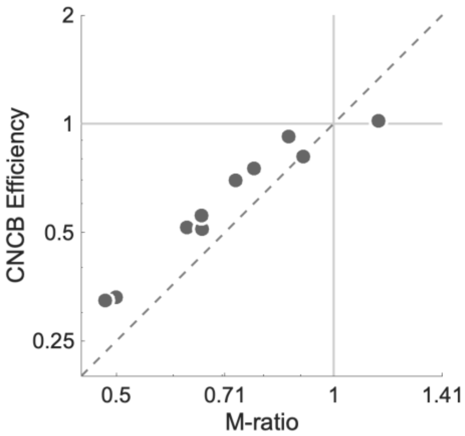

Figure 1. CNCB analysis of Bang et al. [1], Experiment 2, Task 1 (coarse discrimination). […] **(D)** The CNCB efficiency is approximately the square of the M-ratio.

4. I struggled to map some of the results to the conclusions. In particular, on Line 250 (when showing how confidence noise and boost can be recovered with any number of confidence levels) it’s written:

"Unexpectedly, the estimation of these parameters is also possible even when there are only two confidence levels".

Perhaps I’m misreading the figures, but this doesn't seem to be the case for the blue parameters, which at 2 confidence levels are estimated far from the true value (noise of ~1.2 instead of 0.5 and boost of 1 instead of 0.2).

The point we were trying to make here was that confidence noise and confidence boost are not completely random, as it should be theoretically when there are only 2 confidence levels. However, we agree that these parameters are strongly inaccurate and imprecise. We clarified this point in the manuscript (lines 268-275):

As expected, these simulations confirm that confidence noise (**Figure 4B**) and confidence boost (**Figure 4C**) are better estimated when there are more confidence levels. In particular, confidence boost cannot be estimated reliably when there are only two confidence levels (the area under the ROC is below a 75% threshold shown as a dashed line in the upper panel). Unexpectedly, the estimation of confidence noise and boost is not completely random when there are only two confidence levels. We believe that this residual ability comes from small sensory biases inherent of each simulation that create two distinct Type 2 ROC, and that in turn constrain the confidence noise and confidence boost parameters.

Also, the IQRs around the yellow parameters in Fig 2C suggest to me that boost can't be meaningfully estimated for these parameters at all. Likewise, in Fig. 6 (recovering parameters over varying number of difficulty conditions) it seems to me that confidence noise is poorly recovered in most cases for the blue parameters (boost = 0.2, noise = 0.5), and that boost is poorly recovered for all 3 sets of parameters (either because the median is far off the true value, or because the IQR is very large, or both). Assuming I've understood correctly this seems to be a big problem, however if I've misunderstood could the manuscript be updated to clarify what these figures are showing?

We agree with the reviewer that the confidence noise and confidence boost parameters can only be approximately recovered in our simulations. However, distinct values of these parameters can clearly be discriminated. We have added ROC analyses to demonstrate this point, and added these analyses to the relevant figures (see our response above to the 2nd point of Reviewer 1).

Minor comments:

i. Thanks for sharing your toolbox. There's a typo in the doi - from what I could find the toolbox is available at 10.5281/zenodo.13348146, not 10.5281/zenodo.13348147

Thank you for pointing this typo. The number we had included referred to a particular version number. We have now corrected the number to systematically refer to the latest version.

ii. A conceptual description of confidence boost and confidence noise would be really helpful, either in the introduction or results. What kind of psychological processes or manipulations would lead to one or the other increasing/decreasing?

We have clarified the descriptions of confidence noise and boost in the introduction, and added a possible scenario that should increase the confidence boost (lines 88-94).

First, as usually assumed, observers can be specifically impaired in their evaluation of confidence, above and beyond their limited sensitivity in the perceptual decision. This loss of information can be modelled as confidence noise. Second, observers can use information for their confidence judgment that was not used while they computed their perceptual decision. We call this additional information the confidence boost, and note that this component is specific to our approach compared to other work [14]. It is interesting to think how confidence boost could be manipulated experimentally, for instance by varying the delay between Type 1 and Type 2 responses [15].

iii. Eq3: is a unbounded? Fig 2B suggests it's bounded by 0 and 1 but from what I could see it wasn't specifically stated

The parameter α is bounded between 0 and 1. We have clarified this is the text (see line 127).

We call α the boost parameter, which varies between 0 and 1.

iv. Fig 2. Legends are missing. Also Fig 4, 5A, C & D, Figs 6-9, Fig 11

In an attempt to not make the figures too crowded, we prefer not to follow the advice of the reviewer to add legends that would not be easy to make clear (the curves often represent combinations of values of confidence noise and confidence boost). We have nonetheless tried to clarify the figure captions as much as possible.

v. I don't follow Figure 2B. If confidence boost & noise are varying on the x and y axis and all lines are representing participants with matched type 2 HR and FAR, what is being varied here to generate these different coloured lines? Or does each coloured line represent a participant with a different type 2 HR and FAR? If all lines correspond to an observer with a different type 2 HR and FAR, what determines the ideal observer?

We apologize for the lack of clarity for the important Figure 2B. The reviewer is correct is stating that each coloured line represents an observer with different Type 2 hit and false alarm rates. The ideal confidence observer has, by definition, confidence noise and confidence boost equal to 0. But even for this ideal confidence observer, we can define pairs of confidence noise and confidence boost that have identical Type 2 hit and false alarm rates, and these are shown as the black curve.

We have clarified the figure caption for Figure 2B in the manuscript.

(B) Equivalent pairs of confidence noise and confidence boost. Each coloured line represents an observer with a particular set of Type 2 hit and false alarm rates. The blue

curve shows the pairs of confidence noise and confidence boost that are equivalent to the simulated human observer shown by the blue dot that was also the example shown in panel A. The black curve shows the equivalent pairs of confidence noise and confidence boost for the ideal confidence observer (i.e. for whom $\sigma_c = 0$ and $\alpha = 0$). The equivalent confidence noise is obtained when the confidence boost α is set to 1, and is shown as a green dot for the human observer ($\tau_{\mathrm{h}} = 1.39$), and as a black dot for the ideal observer ($\tau_{\mathrm{i}} = 1.60$). The red and yellow curves are equivalent pairs of confidence noise and confidence boost for other simulated observers that are presented later in this paper, namely ($\sigma_c = 0.5, \alpha = 0.8$) in red and ($\sigma_c = 2.0, \alpha = 0.2$) in yellow.

vi. L188-189 Could you clarify what's meant by an 'extreme pair' here?

It is an extreme pair because the parameter α is set to its maximum value. This should be clearer now that we have stated that parameter α is bounded between 0 and 1 (see above).

vii. Typo - L194 says "Below, we compare this CNCB efficiency measure to the M-ratio", but next section doesn't do this. Perhaps sections were ordered differently in an earlier draft?

By “below”, we meant a section below. We have now clarified this point (line 202).

viii. Doesn't the point made on L250 (about being able to recover parameters with only 2 levels of confidence) contradict what's said on L297-299 ("…We restricted the number of confidence levels to 2 (‘high’ and ‘low’) to verify that with only two stimulus strengths, confidence noise and confidence boost cannot be estimated (see Figure 2)."?

As discussed in point 4 above, the confidence noise and boost parameters should not be recoverable in theory. We believe that the fact that they are not completely random in practice comes from small response biases in the simulations.

ix. L270-279 What are the confidence boost & noise parameters estimated from this simulation, and how do they differ when each level of confidence is estimated separately?

The estimated confidence noise and boost are shown in the next figure (Figure 6). We do not understand what the reviewer is asking us to do with each level of confidence, since in these simulations, there are only two confidence levels.

x. L401 "It is known that M-ratios are not fully independent of sensory criteria ([12]; Figure 9B). This is due in part to the fact that when there is a bias in sensory criterion, each sensory strength generates its own Type 2 ROC curve, and the meta-d’ computation needs to find a

way to integrate these two incompatible sets of confidence into a single model." I have several about this:

- Barrett et al claim in that paper that m-ratio on degraded & enhanced signal models (ie when m-ratio ~=1) is effectively invariant to c. So it's true that that reference supports the claim that m-ratio isn't fully independent, but it's not quite true to the spirit of the claim. And indeed, the deviations with c are small (though they're substantial when you analyse response-conditional meta-ds). However when meta-d = d, m-ratio should be invariant to type 1 c by design.

We agree with the reviewer that when meta-d' = d', then the M-ratio (equal to 1) should be invariant to the Type 1 criterion by design. But for any other value of the M-ratio, Figure 6C of Barrett et al. (2013) clearly shows that meta-d' is strongly biased when the Type 1 criterion varies. Our simulations are just a replication of the Figure of Barrett et al., so we think that our reference is justified. We now refer to the corresponding figure of Barrett et al. (lines 460-461):

It is known that M-ratios are not fully independent of sensory criteria (see Figure 6C in [12]; **Figure 10B**).

- Following from that point, I'm struggling to understand why the blue line in Fig 9B isn't flat. Would the authors be able to speculate? Is the expected value of meta-d here 1?

In the simulations underlying the blue line of Figure 9B, the M-ratio turns out to be slightly larger than 1. So it is normal that this line is not perfectly flat. We have removed the horizontal line at 1 that might have introduced some confusion.

- The IQRs for Fig 9B are missing

The IQRs were there in Figures 9B and 9C, they are just very small.

- Could you clarify what's meant by each sensory strength generating its own type 2 ROC? Do you mean that meta-d has to be estimated for each response class separately?

Yes, by definition, and this can be seen as a weakness of meta-d'. We have added a pointer to some previous work related to this point on lines 463-465:

In fact, since the original work of Maniscalco & Lau [10], there has been other proposals to compute meta-d' (see for instance the definition of $\widetilde{d}'_b$ in [12]).

- I think that in its current form this sentence is somewhat misleading, because while it's true that the meta-d model is over-specified (causing this issue with calculating response-conditional meta-ds), CNCB is over-specified too (as shown in Fig 2). Could the authors explain why, despite both models being over-specified, CNCB doesn't end up with a bias but

m-ratio does?

To be perfectly honest, the CNCB efficiency also shows some biases with variations of Type 1 criterion, but they are smaller than those obtained with the M-ratio. The reason why the CNCB model is more robust is precisely because this model is a generative process model, so the Type 1 criterion is an integral part of this model. We have updated the manuscript on lines 465-467:

In contrast, the CNCB model naturally takes into account the sensory criterion because there is only one set of sensory parameters that tries to explain all the perceptual responses and all the confidence judgments (see Eq. 3).

xi. To ensure that the results aren't partly an outcome of how the data were simulated it would be helpful to see how CNCB efficiency and m-ratio covary, and vary with type 1 bias/sensitivity on real data.

Figure 8 in the original manuscript showed how CNCB efficiency covaried with the M-ratio for a small set of parameters. This figure has been updated to Figure 12 to show how these two measures covary over a wider range of variations of the parameter space, including over a ranger of Type 1 sensitivity and bias.

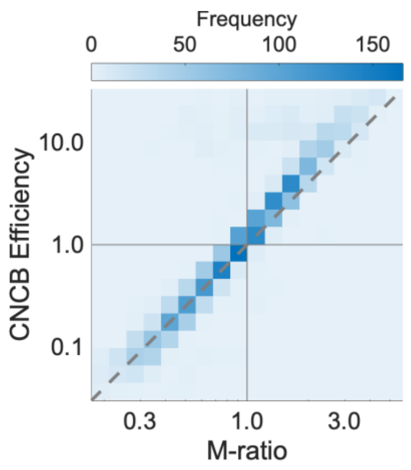

Figure 12. Relationship between confidence efficiency and M-ratio. **(A)** The heatmap shows CNCB efficiencies and M-ratios from 2,500 simulated experiments where there were only two stimulus strengths and four confidence levels. Other parameters were sampled from the ranges described in the Materials and Methods section. The diagonal shows a squared relationship between M-ratio and confidence efficiency in log-log coordinates (see Eq. 7).

xii. Assuming I haven't missed it, I couldn't see a section on gamma recovery in Methods

Figure 11 in the original manuscript showed the gamma recovery. It is now Figure 14 of the revised manuscript.

xiii. L590-597. I'm not sure what the purpose of this self-consistency section is. It is an interesting idea, but isn't used in the paper as far as I can see.

Since the model described in this manuscript is an application to confidence ratings of the model described in a previous paper (Mamassian & de Gardelle, 2022), and since in this other paper, Type 1 performance was evaluated as self-consistency rather than correctness, we feel that it is important to keep this section to make the link.

xiv. I would have liked to see more advice for prospective users of the model in the discussion – what kind of data does CNCB efficiency work on, to what extent can a user evaluate effects of some manipulation on boost and noise separately, and are there any paradigm design choices the researcher should make to maximise their chance of getting clean results?

We thank the reviewer for this request. We have added some advice in the discussion on lines 609-613:

We believe that allowing researchers to release the constraints of a single magnitude of difficulty level and of a finite confidence scale when analysing metacognitive sensitivity is an important contribution of the present work. In addition, our approach does not require that separate models be fitted to each response class, and this may be the reason why the CNCB model is more robust to perceptual biases than meta-d'.

and lines 633-636:

In practice, using three confidence levels or four stimulus strengths (i.e. two magnitudes for each response class) seem sufficient. The confidence boost parameter is more difficult to evaluate than the confidence noise, and good estimates seem to require at least 2,000 confidence judgments.
