# Supplementary material for: The confidence-noise confidence-boost (CNCB) model of confidence rating data
Source: PLoS Comput Biol. 2025 Apr 21;21(4):e1012451. doi: 10.1371/journal.pcbi.1012451 (PMC12043244; doi:10.1371/journal.pcbi.1012451)
Supplement: Attachment — Submitted filename: response_letter_250317.pdf [file pcbi.1012451.s004.pdf]

Response to the reviewers

Dear editor,

We would like to thank you and the reviewers for the detailed comments and for offering us the possibility to revise our manuscript. Overall, we believe that we have addressed all the reviewers' comments, and the revised manuscript has clearly improved in clarity.

Reviewers' comments are reproduced below in black, and our replies in blue. Changes in the revised manuscript are also shown in red.

On behalf of the authors,
Pascal Mamassian

--

Dear Dr. Mamassian,

Thank you for submitting your manuscript to PLOS Computational Biology. After careful consideration, we feel that it has merit but does not fully meet PLOS Computational Biology's publication criteria as it currently stands. Therefore, we invite you to submit a revised version of the manuscript that addresses the points raised during the review process.

Please submit your revised manuscript within 30 days May 11 2025 11:59PM. If you will need more time than this to complete your revisions, please reply to this message or contact the journal office at ploscompbiol@plos.org. When you're ready to submit your revision, log on tohttps://www.editorialmanager.com/pcompbiol/ and select the 'Submissions Needing Revision' folder to locate your manuscript file.

Please include the following items when submitting your revised manuscript:

* A rebuttal letter that responds to each point raised by the editor and reviewer(s). You should upload this letter as a separate file labeled 'Response to Reviewers'. This file does not need to include responses to formatting updates and technical items listed in the 'Journal Requirements' section below.
* A marked-up copy of your manuscript that highlights changes made to the original version. You should upload this as a separate file labeled 'Revised Manuscript with Track Changes'.
* An unmarked version of your revised paper without tracked changes. You should upload this as a separate file labeled 'Manuscript'.

If you would like to make changes to your financial disclosure, competing interests statement, or data availability statement, please make these updates within the submission form at the time of resubmission. Guidelines for resubmitting your figure files are available below the reviewer comments at the end of this letter.

We look forward to receiving your revised manuscript.

Kind regards,

Christoph Strauch
Academic Editor
PLOS Computational Biology

Marieke van Vugt
Section Editor
PLOS Computational Biology

**Additional Editor Comments :**

R3 raises a couple of interesting points and requests for clarification that still need to be addressed (R1 and R2's points have all been comprehensively addressed). If the authors can clearly resolve these, the manuscript should be suitable for a minor revision without requiring further review.

**Reviewers' comments:**

Reviewer's Responses to Questions
Reviewer #1: Apologies for the delay in submitting my review. The authors have comprehensively tackled my previous concerns and I have no further comments.

Thank you for all your comments that helped improving our manuscript.

Reviewer #2: The authors have addressed all of my major concerns. I am happy to recommend the manuscript be accepted for publication.

Thank you for all your comments that helped improving our manuscript.

Reviewer #3: Thanks to the authors for their very thorough revisions. The model, results, and the usefulness of this approach are all much clearer to me now. I've only got a few more questions:

1. Thanks for adding that explanation of boost, it's all clear to me now. You wrote that a possible manipulation that might target boost is varying the delay between Type 1 and 2 responses, but would a simpler one be presenting participants with (valid) information about the stimulus identity, or have I misunderstood? From what I gather, alpha is simply "non-sensory information about the stimulus", so in the most extreme case where the experimenter tells the participant what presented stimulus was then boost should be 1.

We thank the reviewer for this suggestion. We have added this example to help provide an intuition to the reader (lines 135-138):

Intuitively, if the experimenter were to reveal the identity of the stimulus to the participant after their perceptual decision but before their confidence judgment, then these confidence judgments would be consistent with a model where the confidence boost is 1.

2. Fig 7: These new figures are really informative. Even in the 1 parameter model (but also in the 2 parameter model), when confidence noise is less than around 0.5, parameter recovery is not great. Boost in particular can deviate a lot from the true parameter value too.

We thank the reviewer for this comment. It is true that the confidence boost parameter is rather difficult to estimate, and in particular, it tends to cling to the extremes of its domain. We have added a note about this point in the manuscript (lines 394-399):

From the simulations of Model 2, we note that the estimated confidence boost tends to cling to the extremes of its domain (i.e. either 0 or 1; **Figure7C**, right panel). While this is a limitation of our model, we believe that in practice, experimenters will be mostly interested in testing whether confidence boost deviates or not from 0 (the condition where confidence is using the same information as the perceptual decision). In the next section, we examine more closely how well confidence noise and confidence boost can be estimated.

I suppose there will be few cases where a researcher would care about the raw values of those recovered parameters. What I expect will matter in most cases is any condition-wise differences in confidence noise/boost can be recovered.

I think a figure illustrating this would be very useful. If you vary how much d', type 1 threshold or confidence noise changes between 2 conditions and keep all other parameters constant, does the difference in the recovered parameters correlate with the difference in the true parameters? I think the answer should be 'yes' but it'd be nice to see this.

Regarding the suggestion to make a new figure to illustrate that if we simulate 2 conditions, the difference in the recovered parameters should correlate with the difference in the true parameters, we believe that the information is already contained in Figure 8. For instance, Figure 7 and Figure 8B show how well confidence noise is estimated for different simulated values, and indeed, apart from very small values of confidence noise when confidence boost is also small, there is a monotonic relationship between simulated and recovered parameters, and this will generate the correlation that the reviewer is referring to. Therefore, and given the already large number of Figures in this manuscript, we preferred not to add such a new figure.

3. On the topic of parameter recovery, in the results I don't think there's sufficient transparency about the difficulty in recovering boost. There are often statements such as "The simulations shown in Figure 11 indicate that the recovery of confidence noise and

boost is quite robust over a large 500 range of confidence biases" (L 498-500), but the graphs aren't reflecting that. For example, boost for the red parameters in Fig 11D is systematically underestimated and is poorly recovered for the yellow parameters. I think it's important to be clear about the limitations of the model. In any case, despite boost being hard to recover CNCB efficiency behaves really well and confidence noise, mostly well.

We agree that the wording in this sentence may suggest that confidence boost is well recovered overall, irrespectively of confidence bias. Our intention was however to say that the recovery of confidence boost is not affected by confidence bias. We apologize for the confusion, and we have changed the text to clarify this point. We changed the corresponding sentence to (lines 511-513):

The simulations shown in **Figure 11** indicate that the recovery of confidence noise and boost is largely unaffected when introducing confidence bias.

4. On my initial reading of the paper I didn't sufficiently appreciate the final section on continuous confidence ratings. Unless I am missing something, parameter recovery really benefits from using continuous confidence ratings - it seems that it's with continuous ratings that the parameter recovery gets really good. Perhaps more importantly, that a CNCB user doesn't need to bin their continuous confidence ratings into some arbitrary number of categories is a massive advantage of this model over meta-d. I think it'd be worth emphasising this more in the manuscript because this is a really valuable addition to the metacognition toolkit.

We thank the reviewer for this praise of our analysis of continuous confidence ratings. While it is true that the parameter recovery is very good in this case, this simply comes from the fact that the confidence evaluation has a finer resolution. We also agree that this analysis presents the advantage that it does not force participants to project their confidence into some arbitrary number of categories. We have added a couple of sentences to emphasize this analysis (lines 589-594):

The very good parameter recovery comes from the fact that the confidence evaluation has finer resolution than if confidence was restricted to a small number of confidence levels. However, in practice, it is important to check that when instructed to report their confidence on a continuous scale, participants make an effort to report their confidence judgments precisely, rather than simply using two or three confidence levels ("very sure", "not sure at all", and possibly "something in-between").

We have also updated the abstract (lines 23-25):

We also extend the model to experiments using continuous confidence ratings and describe how the model can be fitted without binning these ratings. The continuous confidence model includes a non-linear mapping between objective and subjective confidence probabilities that can be estimated.

And finally, we have updated the author summary (lines 41-42):

Finally, we extend the model to continuous confidence ratings where participants are not restricted to use a limited set of confidence levels.

Minor comments

i. I agree with R1 that there should be a bit more discussion, either in the introduction or discussion, about alternatives to meta-d. Can your new sentence "We call this additional information the confidence boost, and note that this component is specific to our approach compared to other work [14]." Be elaborated on a bit more

We have expanded the sentence that the reviewer is referring to in the Introduction (lines 94-97):

While our model shares some features with other models, in particular the CASANDRE model [14] that can also fit simultaneously multiple stimulus strengths and that also emphasizes the criticality of a good estimate of sensory noise to compute confidence, the introduction of a confidence boost is specific to our approach.

ii. In my previous review I wrote (comment viii): "Doesn't the point made on L250 (about being able to recover parameters with only 2 levels of confidence) contradict what's said on L297-299 ("…We restricted the number of confidence levels to 2 ('high' and 'low') to verify that with only two stimulus strengths, confidence noise and confidence boost cannot be estimated (see Figure 2)."?

In your reply you wrote: "As discussed in point 4 above, the confidence noise and boost parameters should not be recoverable in theory. We believe that the fact that they are not completely random in practice comes from small response biases in the simulations."

I'm afraid I still don't understand this. Maybe my question was unclear – I was asking why the simulations in Fig 6 were run with only 2 levels of confidence (vs e.g 3 levels) when, at least in theory, it should not be possible to recover them.

We apologize for not understanding the reviewer's point previously. In this part of the manuscript, we describe two strategies to disentangle confidence noise and confidence boost. One strategy is to use more than 2 confidence levels (section on page 9), and the other strategy is to use more than 2 stimulus strengths (section on page 12). In the section that the reviewer is referring to, we were interested in demonstrating that the second strategy worked on its own, and thus this is why we restricted the number of confidence levels to 2.

iii. Typo on Line 464 "…since the original wok of Maniscalco and Lau, there *has [have] been other proposals…"

Fixed, thank you!
